# Automation photometer of Hitachi U–2000 spectrophotometer with RS–232C–based computer

**DOI:** 10.1155/S1463924698000248

**Published:** 1998

**Authors:** K. Senthil Kumar, B. S. Lakshmi, Gautam Pennathur

**Affiliations:** Sub-DIC Biotechnology Information Services Centre for Biotechnology Anna University Madras 600 025 India

## Abstract

The interfacing of a commonly used spectrophotometer, the Hitachi U2000, through its RS–232C port to a IBM compatible computer is described. The hardware for data acquisation was designed by suitably modifying readily available materials, and the software was written using the C programming language. The various steps involved in these procedures are elucidated in detail. The efficacy of the procedure was tested experimentally by running the visible spectrum of a cyanine dye. The spectrum was plotted through a printer hooked to the computer. The spectrum was also plotted by transforming the abscissa to the wavenumber scale. This was carried out by using another module written in C. The efficiency of the whole set-up has been calculated using standard procedures.


Journal of Automatic Chemistry, Vol. 20, No. 6 (November–December 1998) pp. 189–193

### Automation of Hitachi U-2000 spectrophotometer with RS-232C-based computer

**K. Senthil Kumar, B. S. Lakshmi and Gautam Pennathur**

*Sub-DIC Biotechnology Information Services, Centre for Biotechnology, Anna University, Madras – 600 025, India*

*The interfacing of a commonly used spectrophotometer, the Hitachi U2000, through its RS-232C port to a IBM compatible computer is described. The hardware for data acquisation was designed by suitably modifying readily available materials, and the software was written using the C programming language. The various steps involved in these procedures are elucidated in detail. The efficacy of the procedure was tested experimentally by running the visible spectrum of a cyanine dye. The spectrum was plotted through a printer hooked to the computer. The spectrum was also plotted by transforming the abscissa to the wavenumber scale. This was carried out by using another module written in C. The efficiency of the whole set-up has been calculated using standard procedures.*

### Introduction

Over the last few years, laboratory automation has focused on the transfer of digitized data from an analytical instrument to the computer, and has become essential in most of the modern chemical laboratories [1–3]. It allows easy calculations to be performed for special data, e.g. measuring spectral area, rate of reactions, etc. The availability of computers and sophisticated programming languages has largely alleviated this problem [4, 5].

In this article, we describe the interfacing of a Hitachi U-2000 spectrometer with an IBM AT/486 compatible mini-computer using C language. The Hitachi U-2000 is a compact low cost instrument working in the wavelength of 1000–200 nm. The instrument is provided with a standard RS-232C port for easy interfacing [6].

The advantage of using the C language compiler is its superiority in terms of speed. Thus, it is best suited for developing interface modules. The other major factor is portability, wherein programs written in C can be run on any computer with little modification [7–9].

The serial port of the computer is connected to the RS-232C interface. The baud rate can be selected from the following 4800, 1200, 600, 300, 150 and 110 bps. The data format is selected as 7E2 (7 bits + even parity + 2 stop bits) [4, 5].

### Remote control

The following sequential operations are carried out on the computer. The remote control option is selected on the screen, then the instrument is put in the external mode. The computer is set to this mode and the instrument controlled by commands (control bytes) sent from the personal computer. The selection of this mode inactivates all the other keys.

### Hardware

The RS-232C (serial, asynchronous interface) connects an IBM AT/486 system with the spectrophotometer (U-2000 series) in the half duplex mode (data transfer takes place in both directions, but only one at a time). It contains 25 pins, out of which 6 pins only are used for connection. It connects the serial COM port of the computer with the serial port of the spectrophotometer. The pin details and cable connections [10] are shown in figure 1.

The Transistor-Transistor Logic level signal (TTL) ranging from 0 to 5 V emanating from the line driver section (MC 1488) of the computer is converted into an RS-232C signal (ranging from −12 to +12 V). The resulting signal TxDATA from the computer goes to the receiving RxDATA pin of the spectrophotometer, where it gets converted to TTL level by the line receiver (HD 75189). Similarly, the signal sent out from the spectrophotometer is converted, and received by the computer. The pin numbers 1-7, 7-7 are short-circuited for protective and common ground, respectively.

The computer and spectrophotometer are not synchronized at the same clock rate, pin 4 RTS (Request-to-Send) of the computer and 5 CTS (Clear-to-Send) of the spectrophotometer, pin 5 CTS of the computer and pin 4 RTS of the spectrophotometer are short-circuited to start and stop data transmission, which is called 'hardware handshaking' process, but 'software handshaking' (acknowledge to the transmitting section that the transmitted byte has been received properly) cannot be done because the spectrophotometer lacks sophisticated microprocessor functions.

### Software

The software is written as per requirements and specification of the user. It takes care of accessing the medium (Medium Access Control Layer) and other interface functions. The modules have been written in C compiler [11, 12].

### Interface functions

The specification of the interface can be stated as follows.

(1) Protocol: Polling
(2) Link: One-to-one

0142-0453/98 $12 00 © 1998 Taylor & Francis Ltd

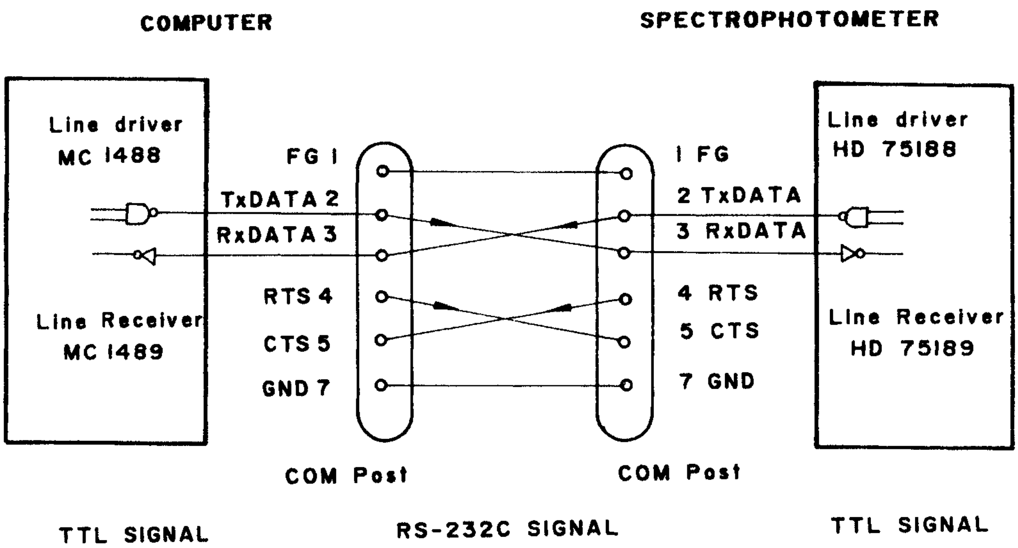

*Figure 1. Cable connections for RS-232C.*

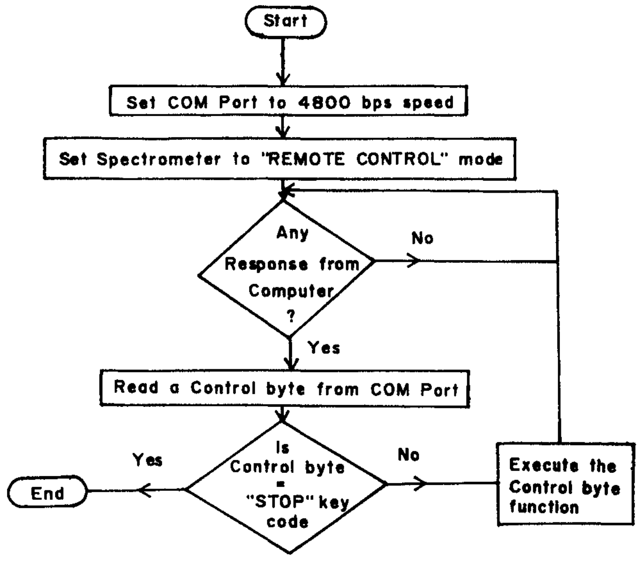

*Figure 2. Spectrophotometer end flowchart.*

(3) Medium: RS-232C(twisted pair)
(4) Speed: 4800 bps
(5) Transmission: Serial, asynchronous and half duples
(6) Distance: ⩽15 m

Baud setting—set data transfer rate of the external system; Status viewing—check whether the spectrophotometer is 'ON' or 'OFF'; Data acquisation—acquire data from the spectrophotometer, history about the received data; Graph plotting is shown in the graph (On-line and Off-line); Peak detection, multiple spectra and area are calculated under the selected portion of the curve.

### Flow chart

The flow charts for interfacing at the spectrophotometer and the computer ends are shown in figures 2 and 3, respectively [9]. The COM port should be set to the desired baud rate. The maximum baud rate is 9600 bps. In order to avoid 'overrun error' and to achieve maximum efficiency in terms of start, stop bits and character length, the medium baud rate is set to 4800 bps. After sending a control byte from the computer to the spectrophotometer, the response is awaited in an indefinite loop. If there is no response or if there is an expectation of a signal from the computer side for the response and a similar one in the spectrophotometer side for the response, then the whole system will be in 'dead lock condition'. As data flows from the spectrophotometer, the computer is made to acquire data and store them in an opened file. After the storage is made, the data are fed as an input to the data processing of the graph plotting phase.

At the computer end of the flowchart, prior to sending the control bytes, the communication port is set at a speed of 4800 bps. Polling (sending some control byte to the receiver section) is done to receive the status of the spectrophotometer. Control signals are sent to the spectrophotometer 1 byte at a time with a proper time delay between 2 bytes (to avoid overrun error) till the control bytes buffer becomes empty. If there are data at the COM port, then the data are received, stored in a file and processed. In the spectrophotometer end of the flowchart, the instrument is set to 'REMOTE CONTROL' mode before setting to 4800 bps speed. If there is any 'response' from the computer, then a control byte is scanned from the COM port. If the 'Control byte' is equal to the 'STOP KEY' code, then terminate the process, else perform the appropriate control byte function (interface function).

### Performance evaluation

### *Analysis*

The evaluation may be done on a time basis. The computer sends a poll byte to the spectrophotometer one at a time to check whether the spectrophotometer is ON or OFF. The poll time taken to send a poll to the spectrophotometer and to receive an acknowledgement

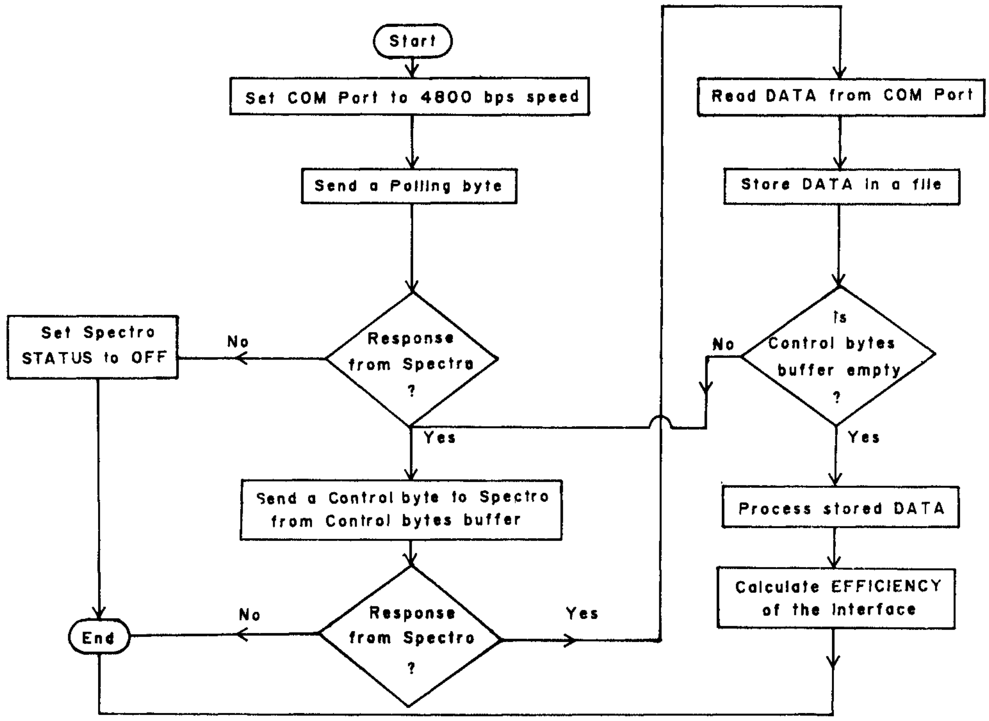

*Figure 3. Computer end flowchart.*

from it is 0.39 ms. The representation of poll exchange on a 4800 bps link between a computer and a spectrophotometer with no data to transmit is shown below

Poll

------------------ >

0.39 ms Response 1

Computer end ----------------- > Spectrophotometer end

Control byte 1

------------------ >

Response 2

------------------ >

A major problem associated with polling is the overhead required in processing the non-productive polls, i.e. polls for which the spectrophotometer has no data to transmit. On this 4800 bps (including overhead) link, it requires approximately 0.01 s of link time to transmit a poll and its response.

### Efficiency

Time taken to send a poll and to receive response = 4.065 ms

Time taken to send a byte = 2.033 ms

The speed in bytes per second = 492 bytes/s or

Speed for data transfer (bps) = 492*8 bps

Each time a byte is sent along with 3 bps (parity, start, stop bits).

In total 11 bits are sent each time.

Efficiency of the interface E = speed/overall baud rate
= 3936/4800
= 82%

### Experimental

The spectrum of a typical cyanine dye 3, 3′-diethylcarboxycyanine iodide was measured. A standard solution of the dye was prepared by dissolving 10 mg of the dye into 25 ml of methanol.

A 1-ml aliquot was further diluted to 10 ml in a standard flask. The solutions were prepared in diffused light. The diluted solution was used for further spectral studies. A pair of matched fused silica cells of path length 1 cm was used for optical measurements.

The spectra of the dye as plotted from the computer to the printer is shown in figure 4. The spectrum wherein the abscissa is transformed into the wave number scale is shown in figure 5.

### Results and discussion

The Hitachi U-2000 is a microprocessor-controlled UV-visible spectrophotometer. The function keys are set in front of the spectrophotometer and the wavelength range, absorbance and other functions can be set through the keyboard.
A major problem associated with polling is the overhead required in processing the non-productive polls, i.e.

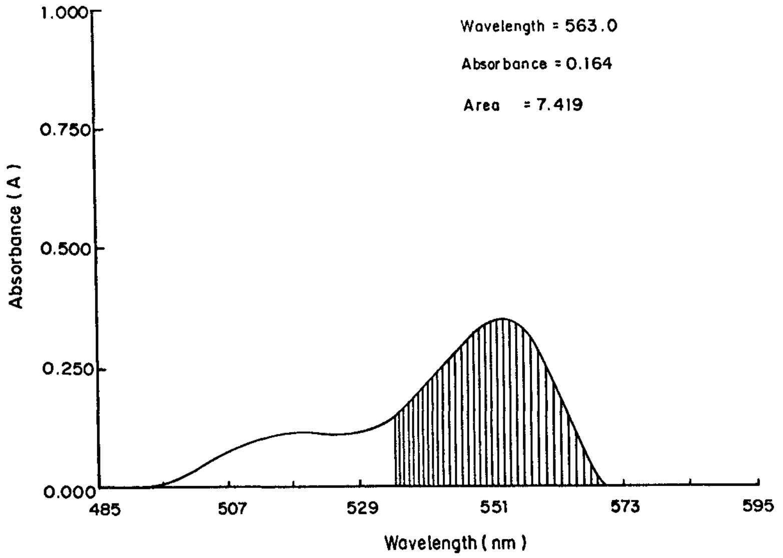

*Figure 4. Wavelength scan of the cyanine dye* ($\lambda_{max} = 563\,\text{nm}$).

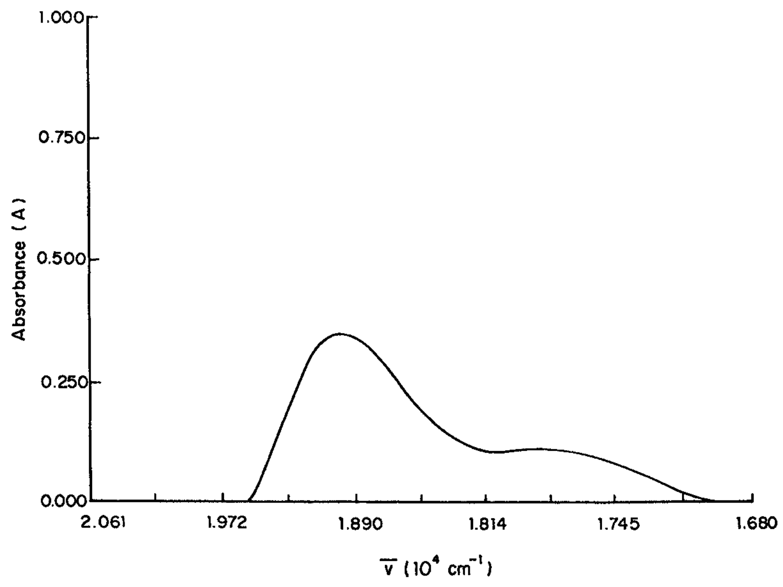

*Figure 5. Plot of wavenumber versus absorbance.*

polls for which the spectrophotometer has no data to transmit.

### Conclusion

The components (ICs, connectors, cables, RS-232C) used here are easily available in the market. The efficiency of this interface is 82%. The interfacing can be done using a range of IBM PC/XT, PC/AT compatibles. Further, this is in dedicated mode, but can be converted to interrupted mode by making the program a Terminate Stay Resident (TSR) program invokable through either software or hardware interrupts. If the keyboard section or printer section of the spectrophotometer fails, those functions are achieved through this alternate interface way of sharing the printer attached to the computer at low cost.

### References

1. Lahiri, S., Yuan, B. and Stillman, M. J., 1994, *Anal. Chem*, **66**, 2954–2962.
2. Korenaga, T., Zhou, X., Morlwake, T., Muraki, H., Naito, T. and Sanuki, S., 1994, *Anal. Chem.*, **66**, 73–78.
3. Glesemann, A., Jager, H. J., Norman, A. L., Krouse, H. R. and Brand, W. A., 1994, *Anal Chem.*, **66**, 2816–2819.
4. Tanenbaum, A. S., 1990, *Computer Networks*, 2nd edition, (Englewood Cliffs, USA: Prentice-Hall inc.).
5. Ahuja, V., 1988, *Design and Analysis of Computer Communication Networks*, 2nd edition.
6. *Hitachi Instruction Manual for Model U-2000 Double Beam Spectrophotometer.*

7. Wang, W. and Bibb, K., 1991, *Illustrated Turbo C++*, Asia-Pacific edition (Texas: Wordware publishing).
8. Horowitz, E. and Sani, S., 1982, *Fundamentals of Data Structures in Pascal* (Maryland: Computer Science Press).
9. Kernighan, B. W. and Ritchie, D. M., 1990, *The C Programming Language*, 2nd edition (Texas: Wordware publishing).
10. Institute of Electrical and Electronics Engineers, 1993, Inc spectrum, Annual review, products and application.
11. Jordan, L. and Churchill, B., 1990, *Communication and Networking for IBM PC/XT Compatibles Revised and Expanded*, (New York: Prentice–Hall).
12. Berry, J. T., 1988, *C++ Programming*, 2nd edition, (Indianapolis: H. W. Sams).

